# Tumor suppressor Spred2 interaction with LC3 promotes autophagosome maturation and induces autophagy-dependent cell death

**DOI:** 10.18632/oncotarget.8357

**Published:** 2016-03-25

**Authors:** Ke Jiang, Min Liu, Guibin Lin, Beibei Mao, Wei Cheng, Han Liu, Jozsef Gal, Haining Zhu, Zengqiang Yuan, Wuguo Deng, Quentin Liu, Peng Gong, Xiaolin Bi, Songshu Meng

**Affiliations:** ^1^ Institute of Cancer Stem Cell, Dalian Medical University Cancer Center, Dalian, China; ^2^ Department of Hepatobiliary Surgery, The First Affiliated Hospital of Dalian Medical University, Dalian, China; ^3^ State Key Laboratory of Brain and Cognitive Sciences, Institute of Biophysics, Chinese Academy of Sciences, Beijing, China; ^4^ Department of Molecular and Cellular Biochemistry, College of Medicine, University of Kentucky, Lexington, Kentucky, USA

**Keywords:** Spred2, LC3, p62/SQSTM1, autophagy, tumor suppressor

## Abstract

The tumor suppressor Spred2 (Sprouty-related EVH1 domain-2) induces cell death in a variety of cancers. However, the underlying mechanism remains to be elucidated. Here we show that Spred2 induces caspase-independent but autophagy-dependent cell death in human cervical carcinoma HeLa and lung cancer A549 cells. We demonstrate that ectopic Spred2 increased both the conversion of microtubule-associated protein 1 light chain 3 (LC3), GFP-LC3 puncta formation and p62/SQSTM1 degradation in A549 and HeLa cells. Conversely, knockdown of Spred2 in tumor cells inhibited upregulation of autophagosome maturation induced by the autophagy inducer Rapamycin, which could be reversed by the rescue Spred2. These data suggest that Spred2 promotes autophagy in tumor cells. Mechanistically, Spred2 co-localized and interacted with LC3 via the LC3-interacting region (LIR) motifs in its SPR domain. Mutations in the LIR motifs or deletion of the SPR domain impaired Spred2-mediated autophagosome maturation and tumor cell death, indicating that functional LIR is required for Spred2 to trigger tumor cell death. Additionally, Spred2 interacted and co-localized with p62/SQSTM1 through its SPR domain. Furthermore, the co-localization of Spred2, p62 and LAMP2 in HeLa cells indicates that p62 may be involved in Spred2-mediated autophagosome maturation. Inhibition of autophagy using the lysosomal inhibitor chloroquine, reduced Spred2-mediated HeLa cell death. Silencing the expression of autophagy-related genes ATG5, LC3 or p62 in HeLa and A549 cells gave similar results, suggesting that autophagy is required for Spred2-induced tumor cell death. Collectively, these data indicate that Spred2 induces tumor cell death in an autophagy-dependent manner.

## INTRODUCTION

Sprouty-related EVH1 domain (Spred) proteins function as negative regulators of growth factors, cytokines and chemokine-induced ERK (extracellular-regulated kinase) activation by binding to Ras and Raf-1 [1–3]. Spreds (Spred1, 2 and 3) contain an N-terminal Ena/VASP homology 1 domain (EVH1), a central c-Kit binding domain (KBD) and a C-terminal cysteine-rich Sprouty-related domain (SPR) that is shared with the Sprouty proteins [1, 4]. Several studies, including our previous work, have reported that the EVH1 and SPR domains play critical roles in the Spred-mediated inhibitory effect on ERK activation [1, 3, 5–9], although the KBD region is dispensable [4]. Accumulating evidence indicates that Spreds function as a tumor suppressor in tumor development and progression [10–14]. Expression of Spred1 and/or Spred2 was frequently reduced in hepatocellular carcinoma and prostate cancer [15, 16], while decreased Spred levels were associated with increased tumor invasion and metastasis [16, 17]. Moreover, *in vitro* and *in vivo* models ectopically expressing Spreds led to a decrease in cancer cell proliferation. This may be due to reduced ERK/MAPK activity [2, 16]. The underlying mechanism by which Spreds suppress tumor growth remains to be elucidated.

Macroautophagy (hereafter referred to as autophagy) is a conserved homeostatic mechanism of lysosomal degradation. The hallmark of autophagy is the formation of double- or multi-membrane vesicles in the cytosol called autophagosomes that encapsulate bulk cytoplasm and cytoplasmic organelles. These autophagosomes mature by fusing with the endocytic compartments (e.g. early and late endosomes, multivesicular bodies) and then fusing with the lysosomal compartment to form autolysosomes, in which the cargo is degraded by acidic lysosomal hydrolases [18, 19]. The process is tightly regulated by a set of core autophagy-related (ATG) proteins, including the ubiquitin-like modifier, ATG8. During autophagy, the microtubule-associated protein 1 light chain 3 (LC3), which is the mammalian homologue of yeast ATG8, is converted to lipidated LC3 II and associates with the autophagic membrane. The accumulation of LC3 II and its localization to the autophagosome (puncta dot formation) are generally used as markers for autophagy [20]. Lipidated LC3 II recruits receptors for specific cargo, such as p62 (also known as SQSTM1) [21], neighbor of BRCA1 (NBR1) [22–24] and adaptor proteins that modulate the movement and maturation of autophagosomes [25, 26]. All known autophagy receptor and adaptor proteins contain one or more LC3-interacting region (LIR) motif(s) with the consensus hydrophobic sequence W/Y/F-X-X-I/L/V [21, 27]. Recent studies have shown that several tumor suppressors, such as p53 and PTEN, may induce autophagy-dependent cell death in tumor cells [28, 29], suggesting that autophagy modulation could be a critical mechanism for tumor suppression.

We previously reported that tyrosines 303/343/353 at the SPR domain is essential for Spred2-mediated inhibition of tumor cell growth [8]. In this study, we show that Spred2 induces autophagy-associated tumor cell death by increasing autophagosome maturation. We further demonstrate that Spred2 enhances autophagosome-lysosome fusion by binding to LC3 via two LIR motifs at the SPR domain. Importantly, both the functional LIR and Spred2-associated autophagy are required for Spred2 to induce tumor cell death. Taken together, our study provides new insights into the underlying mechanisms by which Spred2 induces tumor cell death.

## RESULTS

### Spred2 induces autophagy-associated tumor cell death

**Figure 1:**
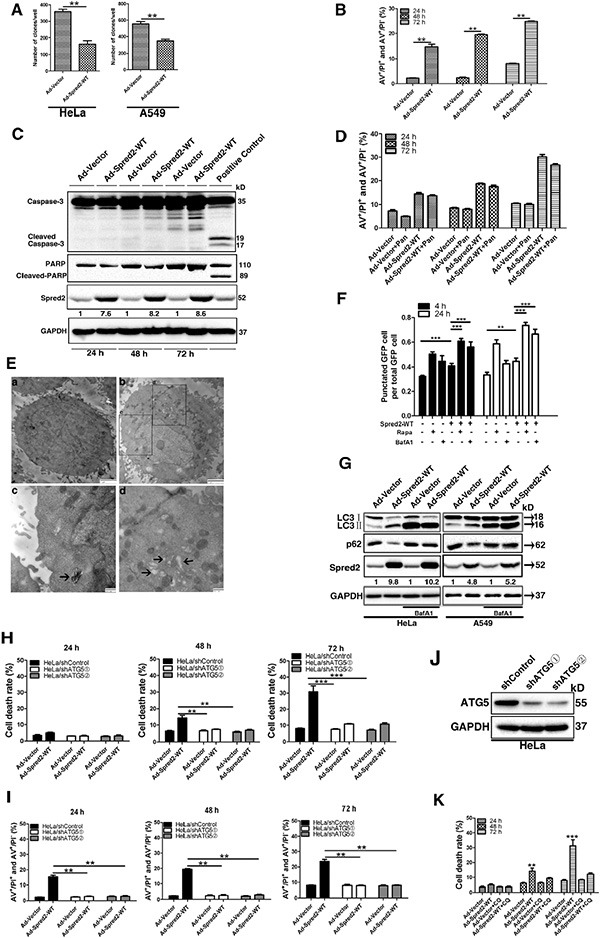
Spred2 triggers autophagy-associated tumor cell death Cells were infected with adenoviruses expressing Myc-Spred2-WT (AdSpred2-WT) or vector control (Ad-Vector) at a multiplicity of infection of 250 for 8 h (A, B, C, E). (**A**) HeLa and A549 cells were cultured in complete medium for 12 days during colony formation assays. (**B**) After 24, 48 or 72 h, HeLa cells were double stained with annexin V (AV) and propidium iodide (PI) for flow cytometric analysis (FACS). (**C**) Cell lysates were analyzed by immunoblotting with anti-Caspase-3, anti-PARP, anti-Spred2 and anti-GAPDH. Doxrubicin was used as a positive control. (**D**) HeLa cells were infected with AdSpred2-WT or Ad-Vector in the presence or absence of pan-caspase inhibitor (50 μM Z-VAD-FMK, Pan) for 24, 48 and 72 h by FACS. (**E**) HeLa cells were examined by transmission electron microscopy (TEM). a, Untreated control HeLa cells showing normal distribution of organelles; b, HeLa cells infected with adenoviruses expressing Spred2. In the lower panels, typical structures enclosed in the black square have been enlarged for more detailed imaging; c, the degradation of the engulfed rough ER is advanced in this degradative autophagosome (indicated by black arrow); d, cup-shaped membranous structures and vacuoles present in the cytoplasm (indicated by black arrows). (**F**) HeLa cells were co-transfected with GFP-LC3 and vector or Myc-Spred2. These were then treated with Rapa (1 μM) or BafA1 (50 μM) for 4 or 24 h. The number of cells with GFP-LC3 punctate are represented. (**G**) HeLa and A549 cells were infected with Ad-vector or Ad-Spred2 and treated with BafA1 (50 μM). Cell lysates were analyzed by immunoblotting for LC3, p62, Spred2 and GAPDH protein expression. Hela/shControl or HeLa/shATG5 cells were infected with Ad-Vector or AdSpred2-WT as indicated for 8 h (H, I). (**H**) After 24, 48 and 72 h, cell death was determined using the trypan blue assay. (**I**) Cell death was determined by AV/PI double staining analysis. (**J**) Cell lysates from HeLa/shControl and HeLa/shATG5cells were analyzed by IB to determine the efficiency of ATG5 knockdown. (**K**) HeLa cells pre-treated with CQ were infected with Ad-Vector or AdSpred2-WT as indicated for 8 h. After 24, 48 and 72 h, cell death was determined using the trypan blue assay. Data are represented as the mean ± S.D from three independent experiments (**p < 0.01, ***p < 0.001).

Using clone formation assays, we showed that infection with adenoviruses expressing Myc-tagged Spred2 (Ad-Spred2) results in the significant inhibition of colony formation in HeLa and A549 cells compared to control virus (Figure 1A), consistent with our previous work and others that Spred2 suppresses tumor cell growth [2, 8, 16]. To investigate whether apoptosis is involved in Spred2-induced tumor cell growth inhibition, HeLa cells infected with Ad-Spred2 were analyzed by flow cytometry using Annexin V and propidium iodide (PI) double-staining. Relative to control virus, Ad-Spred2 infection increased the fraction of cells staining with Annexin V and PI at 24, 48 and 72 h, suggesting that Spred2 may induce apoptosis in these cells (Figure 1B). However, activation of Caspase-3 (effector of apoptosis) and cleavage of PARP (downstream target of active caspase-3) were not observed in Spred2-overexpressing HeLa cells as detected by immunoblotting (Figure 1C). As a positive control, Doxorubicin treatment induced marked cleavage of Caspase-3 and PARP (Figure 1C). Similar results were obtained in A549 cells (Figure S1A). Spred2 levels were modestly overexpressed in these lines by approximately 8-fold relative to the endogenous basal levels (Figure 1C and Figure S1A). Furthermore, Spred2-induced HeLa cell death was not blocked by pre-treatment with the broad-specificity caspase inhibitor, Z-VAD-FMK (Figure 1D), which inhibited Doxorubicin-induced apoptosis (Figure S1B). This would suggest that Spred2-induced cell death may not be caspase-dependent.

Given that autophagy plays a role in cell death triggered by tumor suppressors [28, 29], we hypothesized that Spred2 induces autophagy-associated cell death. To test this, we first examined whether Spred2 induces autophagy in tumor cells. Ultrastructural analysis by transmission electron microscope detected several degradative autophosomes in Ad-Spred2-infected HeLa cells which were rarely observed in HeLa cells infected with control virus (Figure 1E, arrow indicated). To confirm that the observed double-membraned vesicles were indeed related to autophagy, GFP-LC3 dot formation was investigated. Upon autophagy, LC3 is localized on autophagosomes and LC3 puncta are used as a marker for autophagosomes [30]. HeLa cells were co-transfected with GFP-LC3 and Myc-Spred2 or vector control. As depicted in Figure 1F, the number of cells with GFP-LC3 puncta in Spred2-overexpressing HeLa cells was higher than in control cells. Bafilomycin A1 (BafA1) is a specific inhibitor of the vacuolar type H^+^-ATPase (V-ATPase). This is known to block autophagosome-lysosome fusion [31], leading to the accumulation of autophagic vacuoles, as demonstrated by a marked accumulation of LC3II. We observed that BafA1 treatment further increased the number of cells with GFP-LC3 puncta in Spred2-overexpressing HeLa cells (Figure 1F). Similar results were observed in HeLa cells treated with Rapamycin (Rapa), which selectively inhibits mTOR to simulate autophagy (Figure 1F) [30]. The autophagic substrate, p62, which is specifically degraded by the autophagic-lysosomal pathway, was widely used to monitor autophagy flux [32]. Unlike the control cells, in cells with suppressed autophagosome maturation (i.e., BafA1 treated cells) the loss of p62 upon treatment with Ad-Spred2 was not apparent in HeLa and A549 cells (Figure 1G). This BafA1-dependent loss of p62 in response to Spred2 indicates that exogenous Spred2 may activate the formation of autophagosomes rather than suppress their maturation.

To examine whether the autophagy induced by Spred2 serves as a pro-survival or pro-death mechanism, lentivirus expressing shRNA targeting ATG5 were used to stably knock down ATG5 in HeLa and A549 cells (HeLa/shATG5 and A549/shATG5). Atg5 is a key molecule involved in autophagic vacuole formation, and the formation of the Atg12-Atg5 conjugate is indispensable to autophagosome formation. [33–35] So the autophagy process was blocked in ATG5-knockdown cells. Lentiviruses with control shRNA were used to establish the control cell lines (HeLa/shControl and A549/shControl). These cell lines were then infected with AdSpred2 and were analyzed by trypan blue dye exclusion. Spred2-triggered cell death was reduced in HeLa/shATG5 cells compared with HeLa/shControl cells (Figure 1H). This observation was further confirmed by Annexin V and PI double-staining by flow cytometry (Figure 1I). Similar results were obtained in A549/shATG5 cells (Figure S1C and S1D). Depletion of ATG5 in HeLa and A549 cells was confirmed by immunoblotting (Figure 1J and Figure S1E). Consistently, pharmacological inhibition of autophagy by pretreatment with chloroquine (CQ), an autophagolysosome fusion inhibitor [30], attenuated Spred2-induced cell death in HeLa cells (Figure 1K). Together, these data indicate that inhibition of autophagy attenuates Spred2-mediated cell death in tumor cells.

### Spred2 promotes autophagosome maturation in cancer cells

**Figure 2:**
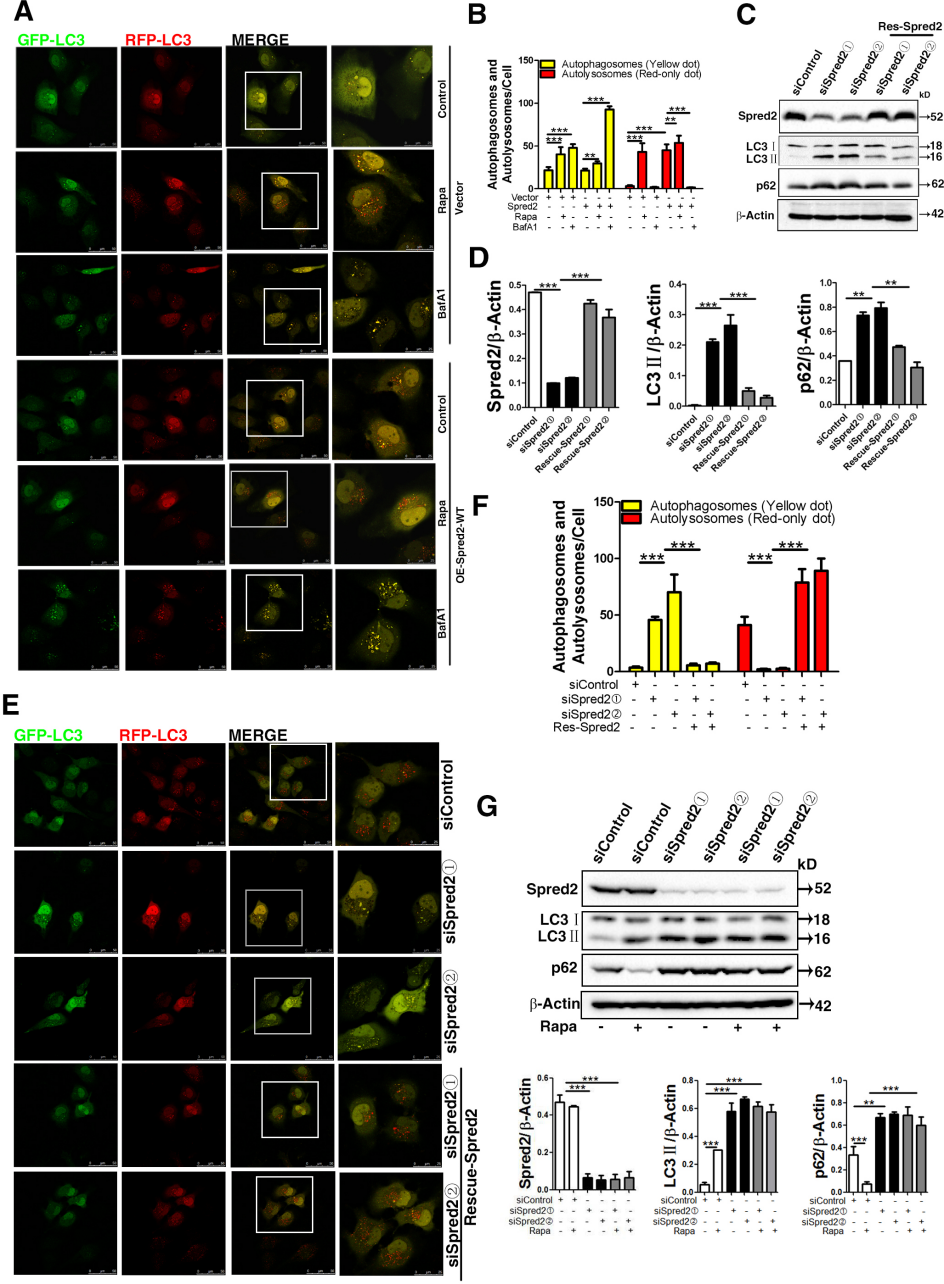
Spred2 enhances autophagosome maturation (**A**) HeLa cells were co-transfected with GFP-mRFP-LC3 and vector or Myc-Spred2 and treated with or without Rapa (1 μM) or BafA1 (50 μM). Images were acquired by confocal microscope. The merged region enclosed within the white square has been enlarged in the right panel for clearer appreciation of these co-localizations. (**B**) The number of autophagosomes (yellow dots) and autolysosomes (red-only dots) per cell were quantified. HeLa cells were transfected with two siRNA duplexes targeted to Spred2 (siSpred2) or control siRNA (siControl) for 48 h (C, D, E, F,). (**C**) Cells were transfected with vector control or a Spred2 rescue plasmid (Rescue-Spred2). After 24 h, cell lysates were analyzed by immunoblotting for Spred2, LC3, p62 and β-actin protein expression. (**D**) The ratios of expression for each protein to their corresponding β-Actin are represented. (**E**) Cells were co-transfected with GFP-mRFP-LC3 and vector control or a Spred2 rescue plasmid for 24 h following Rapa-treatment for 4 h. Images were acquired by confocal microscopy. The merged region enclosed within the white square has been enlarged in the right panel for clearer appreciation of these co-localizations. Scale bars are representative of 50 μm. (**F**) The number of autophagosomes (yellow dots) and autolysosomes (red-only dots) per cell were quantified. (**G**) HeLa cells were transfected with two siRNA duplexes targeted to Spred2 (siSpred2) or control siRNA (siControl) for 72 h. Cells were treated with or without Rapa (1 μM) for 4 h. Cell lysates were analyzed by immunoblotting for Spred2, LC3, p62 and β-Actin protein expression. The ratios of each specific protein to their corresponding β-Actin are represented. All quantitative data are represented as the mean ± S.D from three independent experiments (**p < 0.01, ***p < 0.001).

To elucidate the role of Spred2 in enhancing the autophagic process, we utilized a tandem fluorescent GFP-mRFP-LC3 marker to analyze autophagosome maturation [36]. In this system, autophagosomes display both green and red fluorescence, while autolysosomes display only red fluorescence as the GFP loses its fluorescence from deprotonation in the acidic lysosomes. Therefore, autophagosomes appear as RFP^+^GFP^+^ (yellow dot) and mature, autolysosomal organelles appear as RFP^+^GFP^−^ (red-only dot). Thus, autophagosome maturation can be indicated by a dramatic increase in the number of red-only autolysosomes [36]. HeLa cells were co-transfected with GFP-mRFP-LC3 and Myc-Spred2-WT or vector control. An increase in red-only dot was observed in HeLa cells transfected with Myc-Spred2 (Figure 2A). Moreover, in Spred2-overexpressing cells, treatment with Rapa resulted in a further increase in both yellow and red-only dot, whereas BafA1 treatment led to an increase in yellow dot and decrease in red-only dot (Figure 2A and 2B). In addition, we observed that BafA1-only or Rapa-only treatment increased the number of yellow dot in control HeLa cells (Figure 2A and 2B). As expected, treatment with Rapa induced an increase in the number of red-only dot in control HeLa cells (Figure 2A and 2B). These data suggest that overexpression of Spred2 enhances autophagosome maturation in HeLa cells.

To further establish the role of Spred2 in autophagosome maturation, Spred2 was knocked down by siRNA in HeLa cells and the efficiency was confirmed by immunoblotting (Figure 2C). To rule out the possibility of off-target effects, two independent siRNA oligonucleotides were used, both of which gave similar results. We observed the accumulation of both LC3 II and p62 in Spred2-knockdown HeLa cells but not in control cells transfected with non-target siRNA, suggesting that Spred2 knockdown impaired autophagic degradation (Figure 2C and 2D). In addition, the effect of Spred2 knockdown on LC3 II and p62 levels was rescued by expressing a Spred2 rescue plasmid (Figure 2C and 2D). We also found that Spred2 knockdown could inhibit the upregulation of autophagosome maturation induced by Rapa, which was reversed by the rescue Spred2 expression plasmid (Figure 2E and 2F). This observation would indicate that Spred2 knockdown disrupted autophagosome maturation in cancer cells. Of note, loss of endogenous Spred2 abolished the ability of rapamycin to suppress p62 levels in HeLa cells (Figure 2G), suggesting that Spred2 promotes autophagosomes maturation. Above all, the effect of the autophagy modulators and Spred2 are shown in Table S2.

### Spred2 co-localizes with autophagic organelles

**Figure 3:**
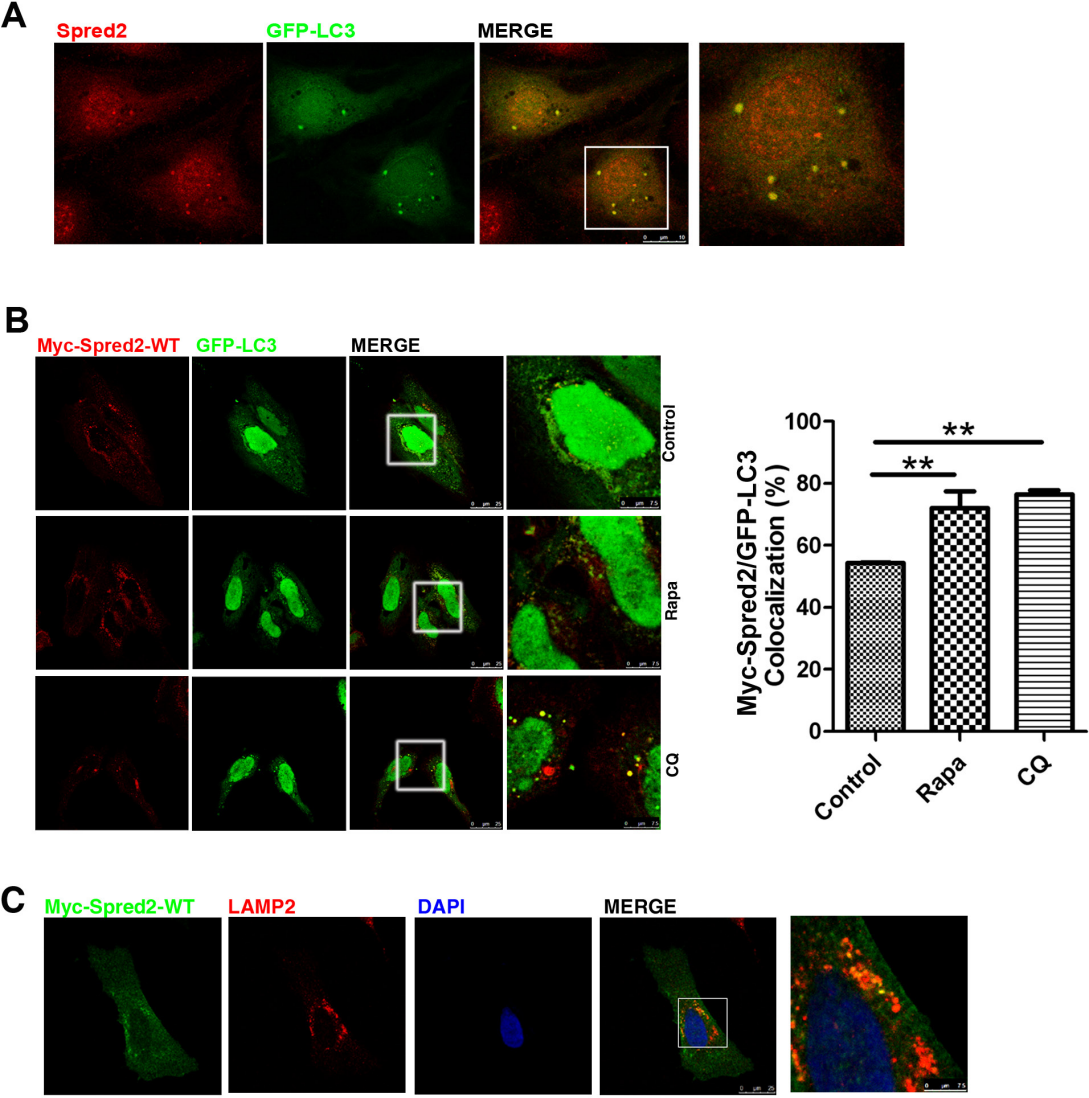
Spred2 localizes with LC3-positive autophagic structures Cells were fixed and analyzed for co-localization by confocal microscopy. The merged region enclosed within the white square has been enlarged in the right panel for a clearer appreciation of the co-localizations. (**A**) HeLa cells overexpressing GFP-LC3 were analyzed for endogenous Spred2/GFP-LC3 co-localization. Scale bars are representative of 10 μm. (**B**) HeLa cells co-transfected with Myc-Spred2 and GFP-LC3 were treated with rapamycin (Rapa, 1 μM), CQ (50 μM), or vehicle control for 4 h. Cells were analyzed for Myc-Spred2/GFP-LC3 co-localization. The percentage of co-localization was quantified. Data are represented as mean ± S.D from three independent experiments (**p < 0.01). (**C**) Myc-Spred2/LAMP2 co-localization in HeLa cells overexpressing Myc-Spred2. Green staining, Myc-Spred2; red, total endogenous LAMP2 and blue staining for the cell nucleus. Scale bars are representative of 25 μm.

The above findings that Spred2 enhances the autophagic process prompted us to explore how Spred2 interacts with the autophagy machinery. We examined whether Spred2 co-localizes with autophagic organelles. ATG16L1 has been used to monitor the movement of plasma membrane as a donor for autophagy, and ATG16L1 is located on phagophores, but not completed autophagosomes.[37, 38] No co-localization was observed between Myc-tagged or endogenous Spred2 and endogenous ATG16L1 in COS7 or HeLa cells. Similarly, CQ did not promote this co-localization (Figure S2A and S2B). However, confocal microscopy analysis revealed substantial co-localization of endogenous Spred2 and GFP-LC3 in cytoplasmic puncta in HeLa cells (Figure 3A). In addition, a similar co-localization of Myc-Spred2 with GFP-LC3 was also observed in HeLa cells (Figure 3B), indicating that Spred2 co-localizes with LC3. We also observed co-localization between Myc-Spred2 and endogenous LAMP2 in HeLa cells (Figure 3C), a late endosomal/lysosomal marker that co-localizes with LC3 during autophagsosome maturation [39]. These data indicate that Spred2 co-localizes with autophagic organelles.

To examine the degree of overlap of Myc-Spred2 and GFP-LC3, each image from three distinct areas (regions of interest, ROIs) were sampled to correct background. Manders' overlap coefficient (R) was employed to evaluate co-localization. An average of coefficients obtained from the examined field was calculated. Rapa treatment increased Myc-Spred2 and GFP-LC3 overlap, whereas treatment with CQ induced co-localization of Spred2 and LC3 on autophagic structures (Figure 3B). Similar results were obtained in COS7 cells (Figure S2C and S2D). These would suggest that the co-localization of Myc-Spred2 and GFP-LC3 were increased by autophagy modulators.

### Spred2 binds to LC3 via the LIR motifs in the SPR domain

**Figure 4:**
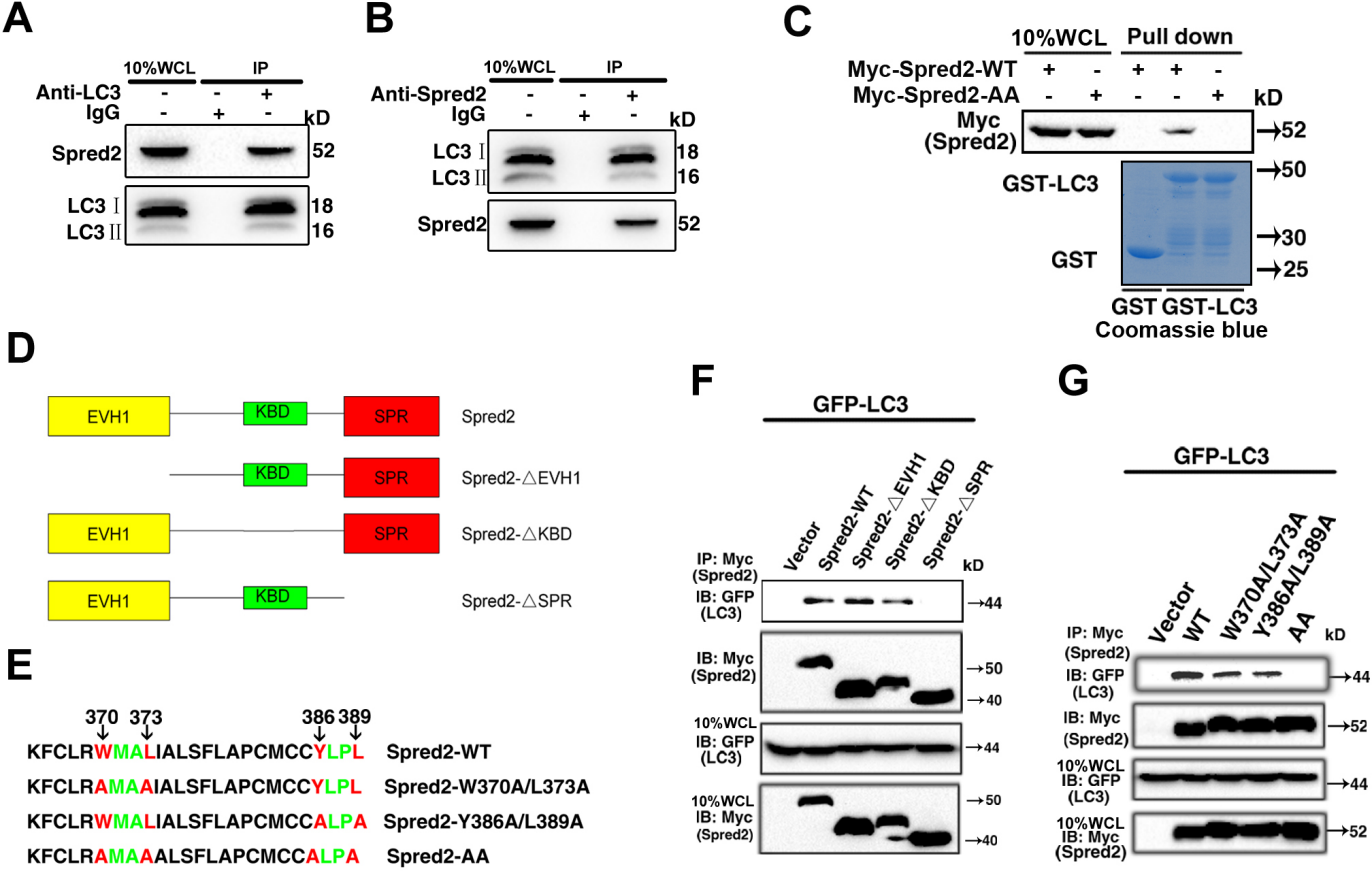
Functional LIR motifs in the SPR domain mediate Spred2/LC3 interaction (**A**) HeLa whole-cell lysates (WCL) collected from 10 cm^2^ dishes were subjected to immunoprecipitation (IP) with an anti-LC3 antibody or an IgG control. (**B**) HeLa whole-cell lysates collected as A were subjected to IP using an anti-Spred2 antibody or an IgG control. (**C**) GST pull-down assays to analyze binding of an *in vitro* translated Myc-Spred2-WT or Myc-Spred2-AA (upper panel) against recombinant human GST-LC3 protein. The purified GST-alone and GST-LC3 protein used are displayed on the Coomassie-stained gels (bottom panel). (**D**) Schematic representation of the Spred2 domains. A series of Myc-tagged truncation constructs of Spred2 were named Spred2-ΔEVH1, Spred2-ΔKBD and Spred2-ΔSPR. (**E**) Sequence alignment of mutations in the SPR domain containing the LC3-interaction region (LIR) motifs (W^370^XXL^373^ and Y^386^XXL^389^). Both tryptophan 370/leucine 373 and tyrosine 386/leucine 389 were mutated to Alanine and the resulting plasmids were subsequently named as Spred2-W370A/L373A and Spred2-Y386A/L389A, respectively. A four mutation of W370/L373 and Y386/L389 was created and named Spred2-W370A/L373A/Y386A/L389A, shown here as Spred2-AA. (**F**) 293T cells were co-transfected with GFP-LC3 and Myc-Spred2-WT or various Spred2 deletion constructs. (**G**) 293T cells were co-transfected with GFP-LC3 and Myc-Spred2-WT or Spred2 mutants. (F, G) Whole-cell lysates (WCL) were IP with an anti-Myc antibody and proteins were resolved by IB. All experiments in this figure were performed as three independent experiments.

The observation of co-localization of Spred2 with LC3 in autophagic organelles led us to speculate whether Spred2 binding to LC3. Endogenous Spred2 and endogenous LC3 were reciprocally immunoprecipitated in HeLa cells (Figure 4A and 4B). The interaction between Spred2 and LC3 was further demonstrated to be direct by *in vitro* pull-down assays with recombinant proteins (Figure 4C), supporting the existence of a Spred2-LC3 interaction at physiological protein levels.

To determine which domain of Spred2 is responsible for this binding, a series of Myc-tagged truncation constructs of Spred2, named Spred2-ΔEVH1, Spred2-ΔKBD and Spred2-ΔSPR (Figure 4D), were prepared and used in co-immunoprecipitation (co-IP) assays. Further motif mapping revealed two potential LIR motifs in the SPR domain; W^370^XXL^373^ and Y^386^XXL^389^ (Figure 4E). GFP-LC3 could be immunoprecipitated with Myc-Spred2 in transfected 293T cells while the SPR domain of Spred2 was the only LC3 binding region (Figure 4F). To determine whether these two LIR sequences in the SPR domain are required for Spred2 binding to LC3, W370, Y386, L373 and L389 were all mutated to alanine (A). The resulting constructs were named as Spred2-W370A/L373A and Spred2-Y386A/L389A, respectively. Surprisingly, mutating either W370/L373 or Y386/L389 alone did not abolish Spred2 binding to LC3. However, four mutations of W370/L373 and Y386/L389 (Spred2-W370A/L373A/Y386A/L389A, shown here as Spred2-AA), completely abrogated LC3 binding to Spred2 (Figure 4G). Similar data was observed in pull-down experiments by Myc-Spred2-AA with GST-LC3 (Figure 4C), indicating that either LIR sequence in the SPR domain is sufficient for Spred2 binding to LC3.

### LIR motifs are required for Spred2-activated autophagosome-lysosome fusion

**Figure 5:**
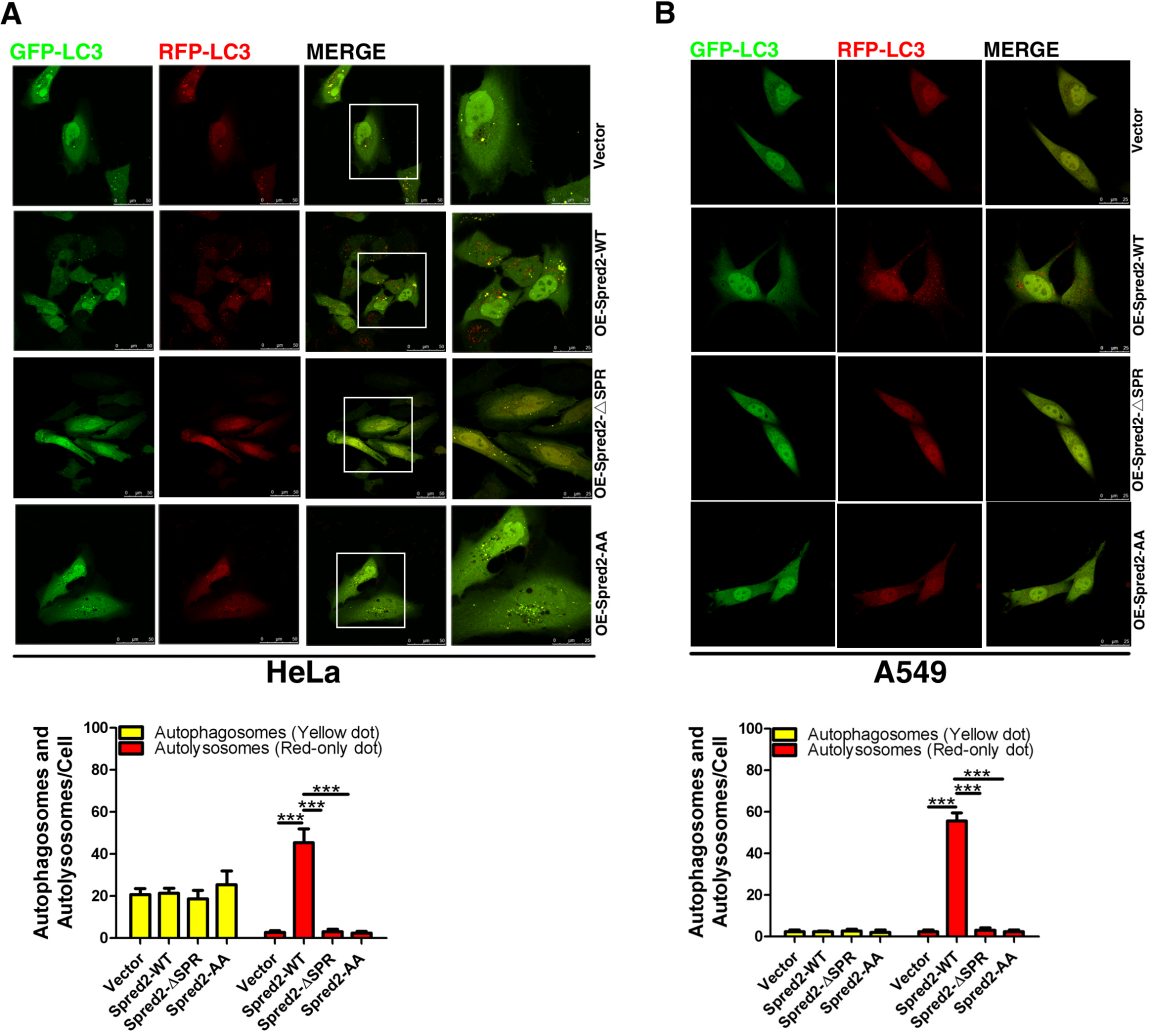
LIR motifs are required for Spred2-mediated autophagosome-lysosome fusion (**A, B**) HeLa cells (**A**) or A549 cells (**B**) were co-transfected with vector, Myc-Spred2-WT, Myc-Spred2-ΔSPR or Myc-Spred2-AA and GFP-mRFP-LC3. Images of live cells were captured by confocal microscopy and the number of autophagosomes (yellow dots) and autolysosomes (red-only dots) per cell were quantified. The merged region enclosed in the white square has been enlarged on the right panel for a clearer appreciation of these co-localizations. Scale bars are representative of 50 or 25 μm. Data are represented as mean ± S.D from three independent experiments (***p < 0.001).

Next, we evaluated whether Spred2 binding to LC3 is essential for its ability to promote autophagosome-lysosome fusion. HeLa cells overexpressing Spred2-WT, Spred2-ΔSPR, Spred2-AA or vector control with the tandem fluorescent GFP-mRFP-LC3 were examined for the level of autophagosome-lysosome fusion. Spred2 overexpression increased the amount of red-only dot representing autolysosomes compared with vector control (Figure 5A). However, there was no change in the amount of red-only dot in cells overexpressing Spred2-ΔSPR or Spred2-AA. Similar results were observed in A549 cells (Figure 5B). Together, these findings indicate that the LIR motifs in the SPR domain are essential for Spred2-activated autophagosome-lysosome fusion.

### Spred2 interacts and co-localizes with p62

**Figure 6:**
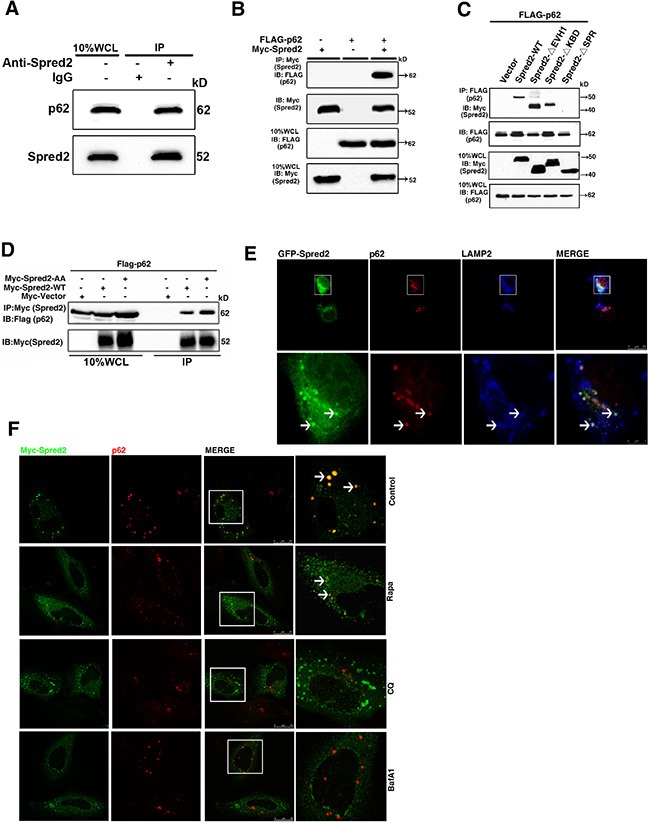
Spred2 interacts and co-localizes with p62 (**A**) HeLa whole-cell lysates collected from 10 cm^2^ dishes were subjected to IP with an anti-Spred2 antibody or an IgG control to detect endogenous Spred2 binding to endogenous p62. (**B**) 293T cells were co-transfected with FLAG-p62 or vector, in addition to Myc-Spred2-WT or vector. (**C**) 293T cells were co-transfected with FLAG-p62 and Myc-Spred2-WT or various Spred2 deletion constructs. WCL were IP with an anti-FLAG antibody. (**D**) 293T cells were co-transfected with FLAG-p62 and Myc-Spred2-AA construct. (B, D) Whole-cell lysates (WCL) were IP with an anti-Myc antibody and were resolved by IB. (**E**) HeLa cells were transfected with GFP-Spred2. The co-localization of GFP-Spred2, endogenous LAMP2 and endogenous p62 was detected. The merged region enclosed within the white square has been enlarged in the bottom panel for better appreciation and the arrows indicate the regions of merge. (**F**) HeLa cells overexpressing Myc-Spred2 were either untreated or treated with Rapa (1 μM), CQ (50 μM) or BafA1 (50 μM) for 4 h. Myc-Spred2/endogenous p62 co-localization was detected. The merged region enclosed within the white square has been enlarged in the right panel for clearer appreciation and the arrows indicate the regions of merge. Scale bars are representative of 25 μm. All experiments in this figure were performed as three independent experiments.

Previously, Heath and colleagues found that Myc-Spred2 interacts with endogenous p62 in 293T cells [9]. Given that Spred2 promotes autolysosome formation, we examined whether p62 is involved in Spred2-mediated autolysosome formation. Endogenous p62 could be immunoprecipitated with endogenous Spred2 in HeLa cells (Figure 6A). In addition, Myc-Spred2 and Flag-p62 were reciprocally immunoprecipitated in 293T cells (Figure 6B and Figure S3A). Furthermore, the binding of Spred2 with p62 *in vivo* was dependent on the SPR domain (Figure 6C), which was confirmed by co-localization experiments (Figure S3B). Notably, Spred2-AA mutant was able to bind and co-localize with p62 in HeLa cells (Figures 6D and S3B). Interestingly, we observed co-localization among GFP-Spred2, endogenous p62 and endogenous LAMP2 in HeLa cells (Figure 6E). The co-localization of Myc-Spred2 and endogenous p62 in HeLa cells was also observed (Figure 6F). This overlap was decreased by CQ or BafA1 (Figure 6F). However, the effect of Rapa on Spred2 and p62 co-localization was minimal (Figure 6F). These data indicate that Spred2 interacts and co-localizes with p62 during the fusion of autophagosomes with lysosomes, a process that is sensitive to CQ and BafA1 treatment.

### LC3 contributes to the interaction and co-localization of Spred2 with p62

**Figure 7:**
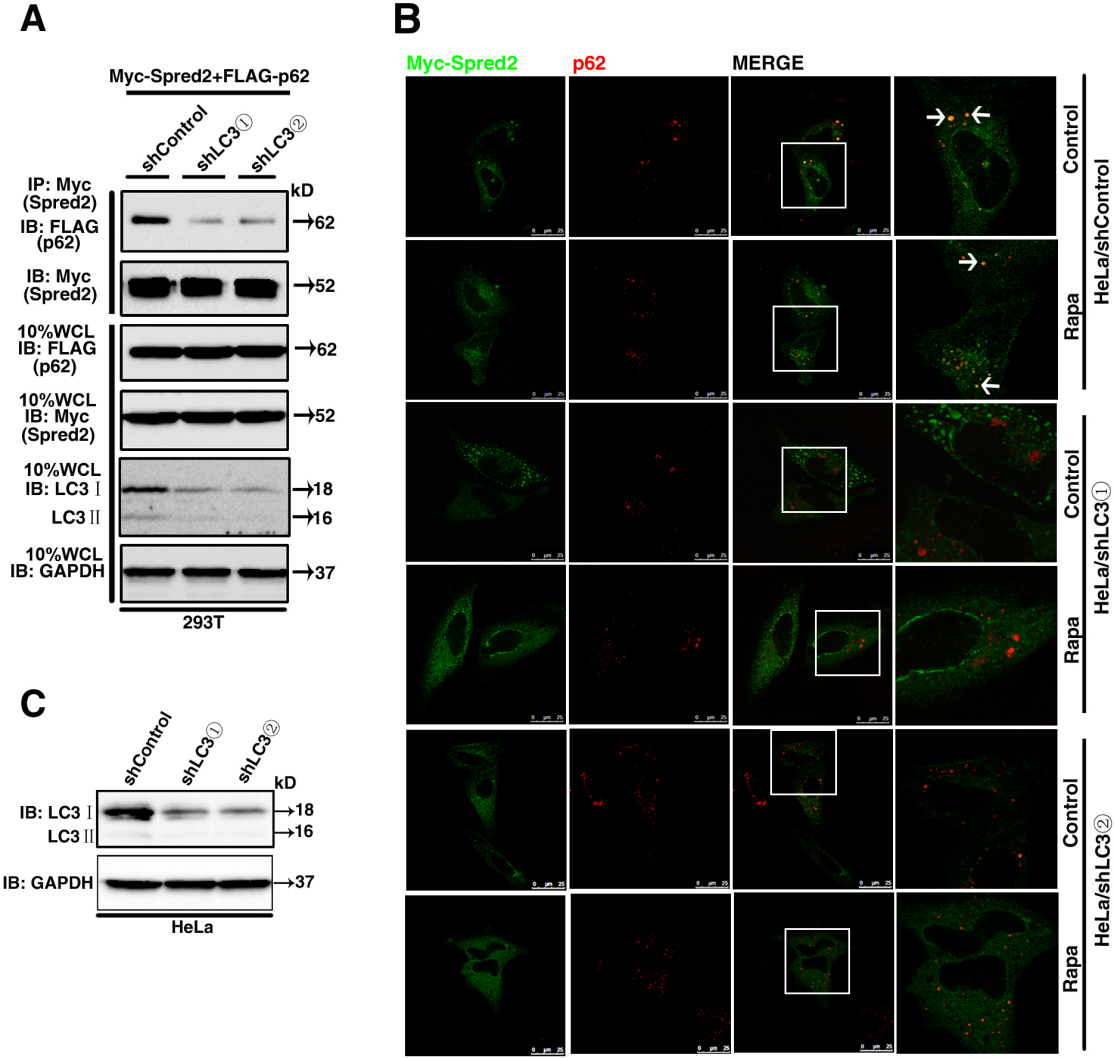
LC3 is essential for Spred2 interaction and co-localization with p62 (**A**) 293T cells with a stable knockdown of LC3 (293T/shLC3) and control cells (293T/shControl) were co-transfected with Myc-Spred2 and FLAG-p62. Whole-cell lysates (WCL) were IP with anti-Myc and resolved by IB. LC3 and GAPDH protein expression in response to LC3 knockdown was determined. (**B**) HeLa cells with a stable knockdown of LC3 (HeLa/shLC3) and control (HeLa/shControl) cells were transfected with Myc-Spred2 and treated with Rapa (1 μM) for 4 h. The co-localization of Myc-Spred2 and endogenous p62 was detected. The merged region enclosed within the white square has been enlarged in the right panel for clearer appreciation and the arrows indicate the regions of merge. Scale bars are represented as 25 μm. (**C**) Cell lysates of HeLa/shLC3 cells and HeLa/shControl cells were analyzed by IB to determine the efficiency of the LC3 knockdown. All experiments in this figure were performed as three independent experiments.

Based on our findings that LC3 is essential for Spred2-mediated promotion on autophagosome maturation, we hypothesized that LC3 is necessary for the binding of Spred2 with p62 during autolysosome formation. To test this hypothesis, Myc-Spred2 and FLAG-p62 were co-transfected in 293T cells with a stable depletion of LC3. Compared with control 293T cells, the binding of Spred2 with p62 was reduced in LC3-knockdown cells, as assayed by co-IP (Figure 7A). Consistently, the co-localization of Myc-Spred2 and endogenous p62 was decreased in HeLa cells with a stable depletion of LC3 compared to control cells (Figure 7B). Inducing autophagy with Rapa did not increase the co-localization of Spred2 and p62 in cells depleted of LC3 (Figure 7B). LC3 protein expression in HeLa cells was determined by immunoblotting (Figure 7C). These results suggest that LC3 is essential for Spred2 interaction and co-localization with p62.

### Abolishment of the association of Spred2 with LC3 and depletion of LC3 or p62 impedes Spred2-mediated tumor cell death

**Figure 8:**
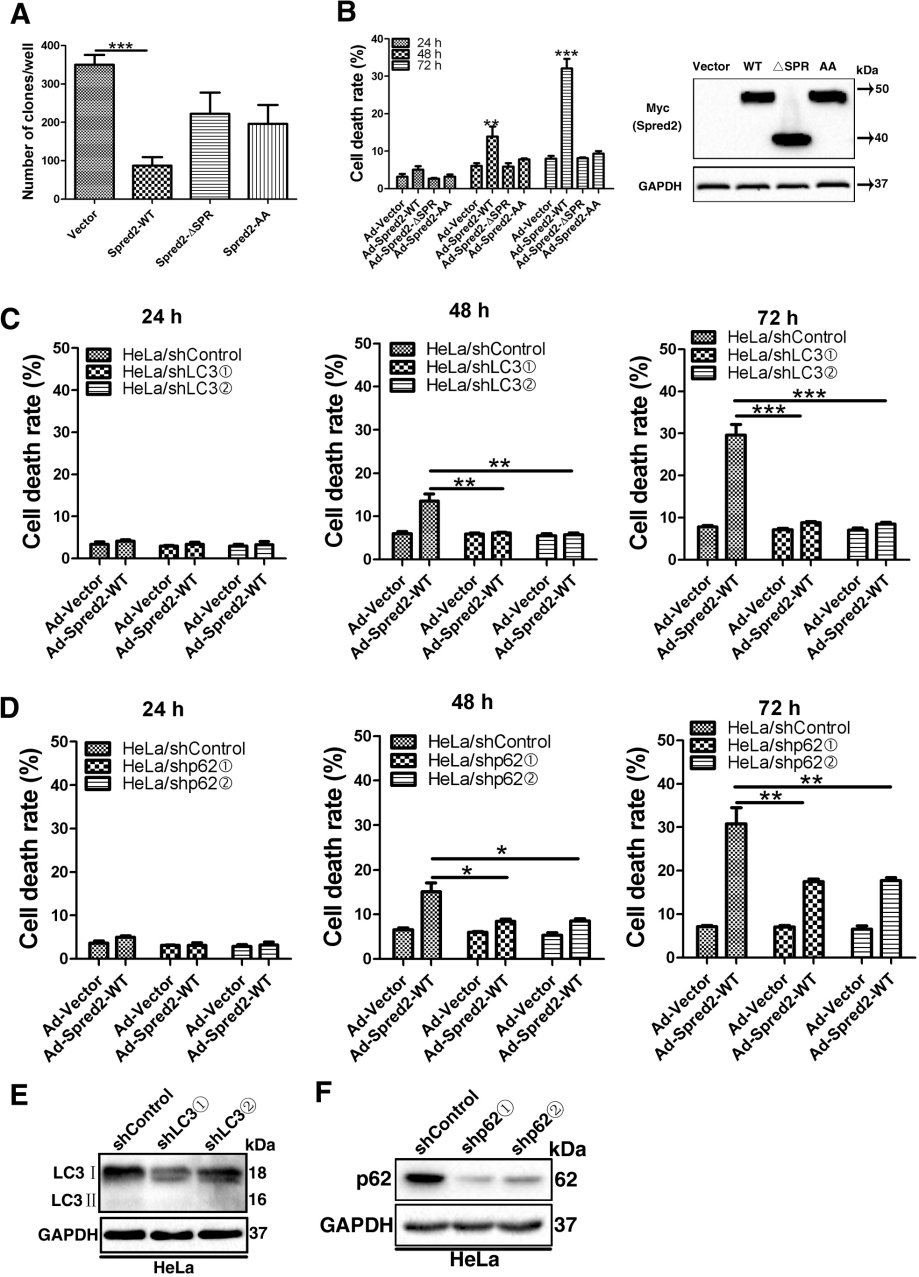
Abolishing the association of Spred2 with LC3 and depletion of LC3 or p62 impedes Spred2-mediated tumor cell death (**A**, **B**) Cells were infected with adenoviruses expressing Myc-Spred2-WT (AdSpred2-WT), Myc-Spred2-ΔSPR (AdSpred2-ΔSPR), Myc-Spred2-AA (AdSpred2-AA) or vector control (Ad-Vector) at a multiplicity of infection of 250 for 8 h. (A) HeLa cells were cultured in complete medium for 12 days during colony formation assays. The graph represents the number of colonies present in the plate at the end of this time. (B) Cell death was determined by cell counting using trypan blue exclusion staining of HeLa cells. Expression in response to the overexpression of constructs was also examined by IB. (**C**, **D**) Cells with a stable knockdown of LC3 (HeLa/shLC3), p62 (HeLa/shp62) or control cells (HeLa/shControl) were infected with adenoviruses expressing Myc-Spred2-WT or vector control at a multiplicity of infection of 250 for 8 h. HeLa/shControl or HeLa/shLC3 cells (C) and HeLa/shControl or HeLa/shp62 (D) were determined by cell counting using trypan blue exclusion staining for cell death analysis. (**E**, **F**) Cell lysates of HeLa/shLC3 cells or HeLa/shControl cells (E) and HeLa/shp62 cells or HeLa/shControl cells (F) were analyzed by IB to determine the efficiency of LC3 or p62 knockdown. Data are represented as mean ± S.D from three independent experiments (*p < 0.05, **p < 0.01, ***p < 0.001).

Given that Spred2 interacts with LC3 and contributes to Spred2-mediated autophagy, we investigated whether the interaction between Spred2 and LC3 is essential for Spred2-induced autophagy-associated cell death. HeLa cells were infected with adenoviruses expressing Myc-Spred2-WT, Myc-Spred2-ΔSPR, Myc-Spred2-AA (LC3 mutated binding sites) or an empty vector control. The cells were then subjected to either a clone formation assay or trypan blue dye exclusion analysis. AdSpred2 infection resulted in the formation of fewer colonies and more dead cells relative to the control virus, AdSpred2-ΔSPR and AdSpred2-AA (Figure 8A and 8B), indicating a role of the LIR in Spred2-induced cancer cell death. The protein levels of these adenoviruses were validated by immunoblot analysis (Figure 8B). To further assess the extent to which the observed cell death depends on autophagy, trypan blue dye exclusion experiments were performed in LC3-knockdown or p62-knockdown HeLa and control cells, infected with AdSpred2 or control virus, respectively. Following AdSpred2 infection, fewer dead cells were detected in p62-knockdown or LC3-knockdown HeLa cells than in control cells (Figure 8C and 8D). We observed similar results in p62-knockdown or LC3-knockdown A549 cells (Figure S4A and S4B). The efficiency of LC3 or p62 knockdown in HeLa and A549 cells was examined respectively by immunoblotting (Figures 8E, 8F, S4C and S4D). Both LC3 and p62 were essential for Spred2-mediated cell death. Together, these data along with results from Figure 1H and Figure 1I, suggest that autophagy contributes to Spred2-induced tumor cell death.

## DISCUSSION

Collectively, our results indicate that Spred2 triggers tumor cell death in an autophagy-dependent manner. We demonstrate that Spred2 induces autophagy and enhances autophagosome maturation in tumor cells. Mechanistically, Spred2 interacts and co-localizes with LC3 via the LIR motifs in the SPR domain, while the functional LIR motifs mediate Spred2-associated autophagosome maturation and contribute to Spred2-induced tumor cell death. In addition, Spred2 interacts and co-localizes with p62 and depletion of p62 in tumor cells, attenuating Spred2-triggered cell death. Together, our data uncover a novel mechanism by which Spred2 exhibits its tumor suppressor function.

To our knowledge, this is the first report showing that autophagy is involved in Spreds-mediated tumor cell death. Our results demonstrated that Spred2, a tumor suppressor, enhances the autophagic process in cancer cells by increasing autophagosome maturation. Furthermore, knockdown of Spred2 impairs the autophagy flux and the autophagy inducer Rapamycin, has no effect on Spred2 knockdown-induced p62 accumulation in cancer cells. These observations indicate that endogenous Spred2 promotes autophagic degradation. Moreover, pharmacological inhibition of autophagy and knockdown of the key autophagy-related genes decreases Spred2-induced cancer cell death, indicating that Spred2 triggers cancer cell death in an autophagy-dependent manner.

We also provide insights into the molecular mechanism by which Spred2 enhances autophagosome maturation. Specifically, our results underline the importance of the interaction of Spred2 with LC3 via the two LIR motifs in the SPR domain. Mutations in the LIR motifs decreased the number of red-only autolysosomes, thereby impairing the autophagosome-lysosome fusion. Previous studies have reported an interaction between Spred2 and p62 [9]. We further demonstrate that the SPR domain mediates the binding and co-localization of Spred2 with p62. Consistent with previous findings by Mardakheh *et al*. [9], we observed that Myc-tagged-Spred2-ΔSPR was mainly localized in the nucleus of cancer cells, while the majority of myc-Spred2 in these cells exhibited a puncta cytoplasmic staining. It is possible that the nuclear localization of Spred2-ΔSPR is either the reason or the consequence of the lacking interaction with autophagosome components. Notably, we found that Spred2 interaction and co-localization with p62 is dependent on the Spred2/LC3 interaction. Spred2 is also shown to interact with NBR1 and of interest, co-localizes with NBR1 and p62 [9]. Both NBR1 and p62 are selective autophagy substrates and receptors that bind to LC3 via their LIR motifs. Based on our observations that Spred2 interacts and co-localizes with LC3 and p62, our data suggest that Spred2 is a scaffold protein in autophagosome maturation.

Spred2 functions as a tumor suppressor in a variety of cancers. Decreased expression of Spred2 is observed in hepatocellular carcinoma and prostate cancer [15, 16], while forced Spred2 expression suppresses cell growth in a variety of tumor cells [15–17]. We recently reported that forced expression of Spred2 inhibits HeLa cell growth while deletion of the SPR domain attenuates this inhibitory effect [8]. In the present study, we show that Spred2-associated autophagy can induce tumor cell death. This notion was further supported by our observation that Spred2-mediated tumor cell death was impaired by either mutations in the LIR motifs in Spred2 or knockdown of ATG5, LC3 or p62. Several reports have shown that tumor suppressors trigger autophagy-dependent cell death [28, 29, 40]. In a study by Seillier *et al.*, the tumor suppressor protein 53-induced nuclear protein 1 (TP53INP1), was shown to bind to LC3 through the LIR and promotes autophagy-dependent tumor cell death [40]. The tumor suppressor p53 also induces autophagic cell death in cancer cells [41]. These data together with our findings, suggest that the autophagy process promoted by tumor suppressors, may be a mechanism in tumor suppression.

Taken together, this study demonstrates that through its interaction with LC3, Spred2 enhances autophagosome maturation thereby contributing to tumor cell death. Our study also suggests that autophagy may be a potential target for enhancing Spred2-mediated anti-tumor activity.

## MATERIALS AND METHODS

### Cell culture and transfection

Human embryonic kidney cells (293T), monkey fibroblast-like kidney cells (COS7), human cervical carcinoma cells (HeLa) and human lung cancer cells (A549) were purchased from American Type Culture Collection (ATCC). 293T, COS7 and A549 cell lines were cultured in DMEM (Gibco) supplemented with 10% fetal bovine serum (FBS) and HeLa cells were cultured in MEM (Gibco) supplemented with 10% FBS. All cells were cultured in a humidified incubator under 5% CO_2_ at 37°C. Transfection of plasmids into all cells (for immunoprecipitation or immunofluorescence) was performed using Lipofectamine 2000 (Invitrogen), according to the manufacturer's instructions.

### Antibodies and reagents

For a list of antibodies, see Table S1. Bafilomycin A1 (BafA1) was purchased from Merck Millipore. Doxorubicin (Dox), Chloroquine (CQ) and Rapamycin (Rapa) were purchased from Sigma and diluted in dimethyl sulfoxide (DMSO). The caspase inhibitor Z-VAD-FMK (Pan) was purchased from Selleckchem. Small interfering RNA (siRNA) against Spred2 and non-targeting siRNA controls were obtained from Qiagen.

### Immunoprecipitation and immunoblotting

Immunoprecipitation (IP) and immunoblotting (IB) were performed as previously described [42]. In some cases, membranes were stripped and re-probed with specific antibodies. To quantify changes, the densitometries of protein bands were determined with a calibrated GS-670 densitometer. For endogenous interactions, HeLa cells grown in 10 cm^2^ dishes were harvested and the cell lysates were then subjected to IP. All IP and IB experiments were performed as three independent experiments.

### GST-pull down assays

GST-tagged-LC3 protein was expressed in *Escherichia coli* SoluBL21. Expression was induced by addition of 0.5 mM IPTG and cells were incubated at 20°C overnight. Collected cells were lysed using sonication in buffer (20 mM Tris-HCl at pH 7.5, 10 mM EDTA, 5 mM EGTA and 150 mM NaCl). GST fusion protein was purified on glutathione-Sepharose 4 Fast Flow beads (GE Healthcare). For GST pull-down using cell lysates, 293T cells were transfected with Myc-Spred2 using transfection reagent (Lipofectamine 2000, Invitrogen). Cells were lysed and were incubated with GST-LC3 protein on glutathione-Sepharose beads in binding buffer (50 mM Tris, 100 mM NaCl, 1 mM EDTA, 0.5% NP-40, pH 8.0,) supplemented with protease inhibitor for 1 h at 4°C. Following five washes, the beads and precipitated proteins were eluted with 5 × SDS-PAGE loading buffer, boiled and loaded on SDS-PAGE gels for analysis.

### Plasmids, adenovirus and lentivirus

Myc-tagged Spred2 and deletion mutants (ΔEVH1, ΔKBD and ΔSPR) were kindly provided by Prof. John K Heath. Mutations for tryptophan 370/leucine 373 and tyrosine 386/leucine 389 were mutated to Alanine in Spred2 and introduced by PCR-based site-directed mutagenesis. The resulting construct was named Spred2-AA. The GFP-tagged Spred2 was constructed per standard molecular cloning procedures. FLAG-tagged p62 was kindly provided by Prof. Haining Zhu. mRFP-GFP-tagged LC3 was kindly provided by Yoshimori [43]. GFP-tagged LC3 was purchased from Addgene. Adenoviruses expressing Myc-tagged Spred2 and Myc-tagged Spred2-ΔSPR and the control virus (Ad-Vector) were previously described [8]. The AdEasy XL adenoviral vector system (Stratagene) was used to generate adenovirus-Myc-Spred2-AA. Primers used were as follows: 5′-GGGGTACCATGACCGAAGAAACACACC-3′ and 5′-GCCAAGCTTATGGTGATGGTGATGATG-3′. The following lentiviral constructs were purchased from Santa Cruz: p62 (SQSTM1) shRNA (sc-29679-V), ATG5 shRNA (sc-41445-V), MAP LC3β shRNA (sc-43390-V) and non-coding shRNA (sc-108080). Lentiviral particles were used to directly infect 293T, A549 and HeLa cells. Stable clones were then selected using puromycin (Sigma). The selected cell populations were subjected to immunoblotting to investigate the silencing efficiency.

### RNA interference

RNA interference was used to knock down targeted proteins of interest. Two siRNA oligonucleotides were used for Spred2: 5′-AAGGACTTGGTCTACACCAAA-3′ and 5′-CAGACCCTTGCTCGTGCGATA-3′. Transfection of siRNA was performed as previously described [42]. A scrambled siRNA was used as a negative control and the silencing efficiency was detected by immunoblot analysis. A Myc-tagged Spred2 rescue plasmid was generated by creating three silent base-pair mutations in the wild type sequence [44].

### Transmission electron microscopy

For ultrastructural analysis, standard transmission electron microscopy (TEM) was carried out. Adenovirus infection was performed as above. Four hours after infection, the cells were fixed and embedded. Thin sections (90 nm) were cut and examined at 80 kV with a JEOL 1200EX transmission electron microscope. Approximately 15 cells were counted and autophagosomes were defined as structures measuring 0.5 to 2.0 μm.

### Fluorescence microscopy and indirect immunofluorescence

For fluorescence microscopy, HeLa cells were transfected with GFP-tagged LC3 using Lipofectamine 2000. Dot formation by GFP-LC3 was detected with a fluorescence microscope (Olympus IX81) as previously described [45]. Transfected cells with five or more puncta were considered to have accumulated autophagosomes. A total of 100 transfected cells were examined per well, in triplicate, from three independent experiments.

For immunofluorescence microscopy, cells were plated on coverslips (NEST, 801008) 24–48 h before immunostaining. Cells were washed twice with ice-cold PBS and were fixed in 4% paraformaldehyde (PFA) for 30 min, permeabilized in 0.2% Triton X-100 for 15 min and incubated for 60 min in 3% Bovine Serum Albumin (BSA). Cells were then incubated with primary antibody overnight followed by a secondary antibody in PBS containing 3% BSA. Nuclei were stained with 5 μg/mL DAPI (Sigma) in PBS. Images were acquired using a confocal microscope (Leica TCS SP5 ×) with a 60 × oil objective. Images from each experiment were acquired using the same exposure time during the same imaging session. Quantification of autophagic vacuoles was analyzed by calculating the number of LC3 puncta from three fields containing more than 5 randomly selected microscopy-captured images, each comprising between 2 and 8 cells. Autophagosomes were detected as RFP^+^GFP^+^ (yellow dot), while mature, autolysosomal organelles were detected as RFP^+^GFP^−^ (red-only dot) [46–48]. To examine the degree of overlap of red (Myc-Spred2) and green (GFP-LC3) spots, analysis of pixel co-localization was performed using the co-localization function of Image-Pro Plus software. The gain and offset were lowered to prevent saturation in the brightest signals. From each image three distinct areas (regions of interest, ROIs) were sampled to correct for background. Manders' overlap coefficient (R) was employed to evaluate co-localization, and therefore could be used to determine the co-localization of two signals. The values of Manders' overlap co-efficientare are in the range of 0 to 1.0. If the image has an overlap coefficient of 1, it implies that 100% of both selected channels overlap or co-localize. A value of zero means that there are no overlapping pixels. It can be used in any co-localization experiment. To assess the reliability of this method, we applied the same procedure to HeLa and COS7 cells for the co-localization of Myc-Spred2 and GFP-LC3. Quantification of the co-localization was analyzed from three fields containing more than 5 randomly selected microscopy-captured images, each of which comprised at least two cells. For each area an average of coefficients obtained from the examined field was calculated. The *t*-test was used to assess the results [49].

### Flow cytometry analysis of apoptosis

Apoptosis was determined by flow cytometric analysis of membrane re-distribution of phosphatidylserine using Annexin V and propidium iodide (PI) double-staining technique. The cell population in the upper right and lower right quadrants (Annexin V-positive/PI-positive and Annexin V-positive/PI-negative, respectively) corresponds to apoptotic cells. The percentage of apoptotic cells was determined in three independent experiments.

### Trypan blue exclusion assay

Cells were seeded at 3 × 10^4^ cells/well in 12-well plates. The following day, cells were infected with adenoviruses at a multiplicity of infection (MOI) of 250. The trypan blue exclusion assay was performed to determine the level of cell death as previously described [8]. Experiments were repeated at least three times.

### Colony formation assay

Cells were cultured in complete medium supplemented with 10% FBS at 37°C in 5% CO_2_. The colonies (containing 50 or more cells) were counted by light microscopy after 12 days. All semi-solid cultures were performed in triplicate. Three independent experiments were performed.

### Statistical analysis

Data were first evaluated using one-way analysis of variance (ANOVA). Multiple comparisons between treatment groups and controls were performed using Dunnett's least significant difference (LSD) test. Statistical significance between groups was calculated using the LSD test in SPSS 17.0 software (SPSS Inc., Chicago, IL, USA). A value of *p* < 0.05 was considered statistically significant.

## SUPPLEMENTARY MATERIALS FIGURES AND TABLES



## Abbreviations

Spred: Sprouty-related protein with an EVH1 domain
EVH1: Ena/VASP homology 1
KBD: c-Kit binding domain
SPR: Sprouty-related domain
LC3: microtubule-associated protein 1 light chain 3
SQSTM1: sequestosome 1
LIR: LC3-interacting region
Rapa: rapamycin
CQ: chloroquine
BafA1: Bafilomycin A1
siRNA: small interfering RNA
shRNA: short hairpin RNA
IP: immunoprecipitation
IB: immunoblot
