# Supplementary material for: Tumor suppressor Spred2 interaction with LC3 promotes autophagosome maturation and induces autophagy-dependent cell death
Source: Oncotarget. 2016 Mar 25;7(18):25652–67. doi: 10.18632/oncotarget.8357 (PMC5041934; doi:10.18632/oncotarget.8357)
Supplement: Supplementary file 1 [file oncotarget-07-25652-s001.pdf]

Tumor suppressor Spred2 interaction with LC3 promotes autophagosome maturation and induces autophagy-dependent cell death

Supplementary Materials

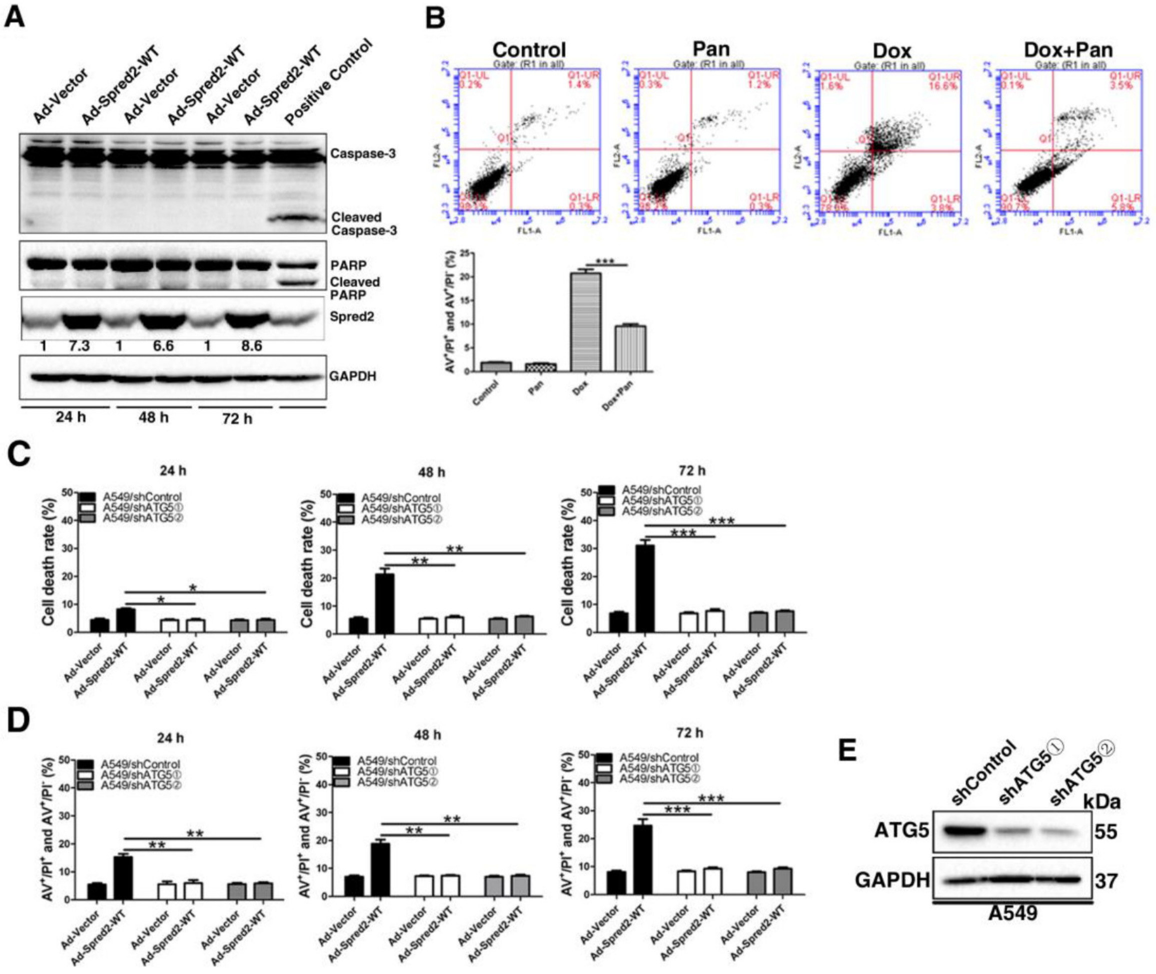

Supplementary Figure S1: Spred2 triggers autophagy-associated cell death in A549 cells. (**A**) A549 cells were infected with adenoviruses expressing Myc-Spred2-WT (AdSpred2-WT) or vector control (Ad-Vector) at a multiplicity of infection of 250 for 8 h, and cell lysates were analyzed by immunoblotting with anti-Caspase-3, anti-PARP, anti-Spred2 and anti-GAPDH. Doxrubicin was used as a positive control. (**B**) HeLa cells were treated Doxorubicin (DOX) in the presence or absence of caspase inhibitor 50 μM Z-VAD-FMK (Pan) for 24 h, cells were double stained with annexin V and PI for FACS. A549 or A549/shATG5 cells were infected with Ad-Vector or AdSpred2-WT as indicated for 8 h (C, D). (**C**) After 24, 48 and 72 h, Cell death was determined using trypan blue assay. (**D**) Cell death was determined by AV/PI double staining analysis. (**E**) Cell lysates from A549/shATG5 cells and A549/shControl cells were analyzed by IB to determine the efficiency of the ATG5 knockdown. Data are represented as mean ± S.D from three independent experiments (*$p < 0.05$, **$p < 0.01$, ***$p < 0.001$).

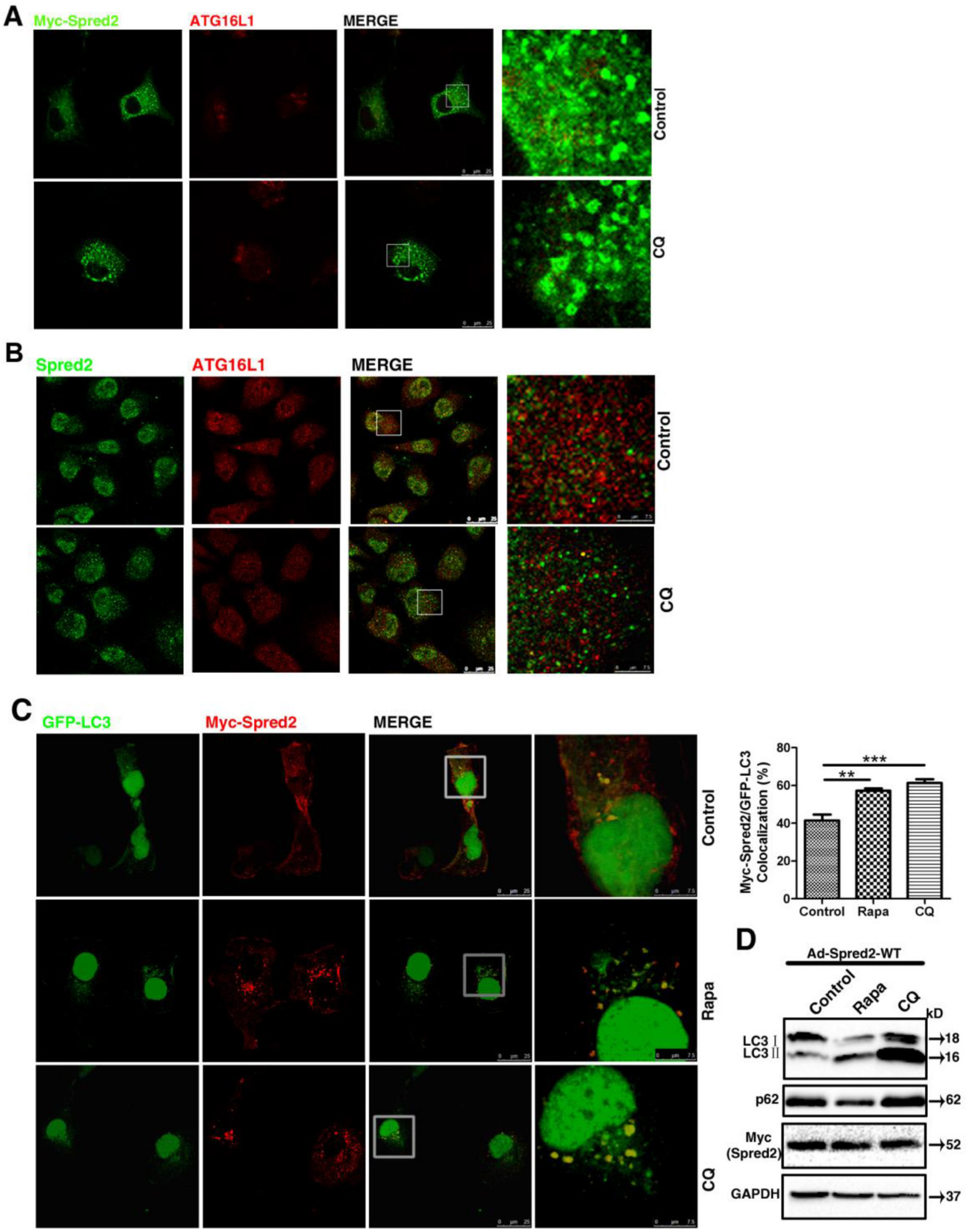

Supplementary Figure S2: Spred2 co-localizes with LC3. Cells were fixed and analyzed for co-localization by confocal microscopy. The merge region enclosed within the white square has been enlarged in the right panel for a clearer appreciation of colocalizations (A, B, C). (**A**) Overexpressing Myc-Spred2 in COS7 cells which were untreated or treated with CQ (50 μM) for 4 h and were analyzed for Myc-Spred2/ATG16L1 co-localization. (**B**) Co-staining of endogenous Spred2 (green) and endogenous ATG16L1 (red) in HeLa cells which were untreated or treated with chloroquine (CQ, 50 μM). COS7 cells co-transfected with GFP-LC3 and Myc-Spred2 were treated with rapamycin (Rapa, 1 μM), chloroquine (CQ, 50 μM), or vehicle for 4 h (C, D). (**C**) Cells were analyzed for GFP-LC3/Myc-Spred2 co-localization. The percentage of co-localization was quantified. Data are represented as mean ± S.D from three independent experiments (**$p < 0.01$, ***$p < 0.001$). Scale bars represent 25 μm. (**D**) Cell lysates were analyzed by immunoblotting with anti-LC3, anti-p62, anti-Spred2 and anti-GAPDH antibodies.

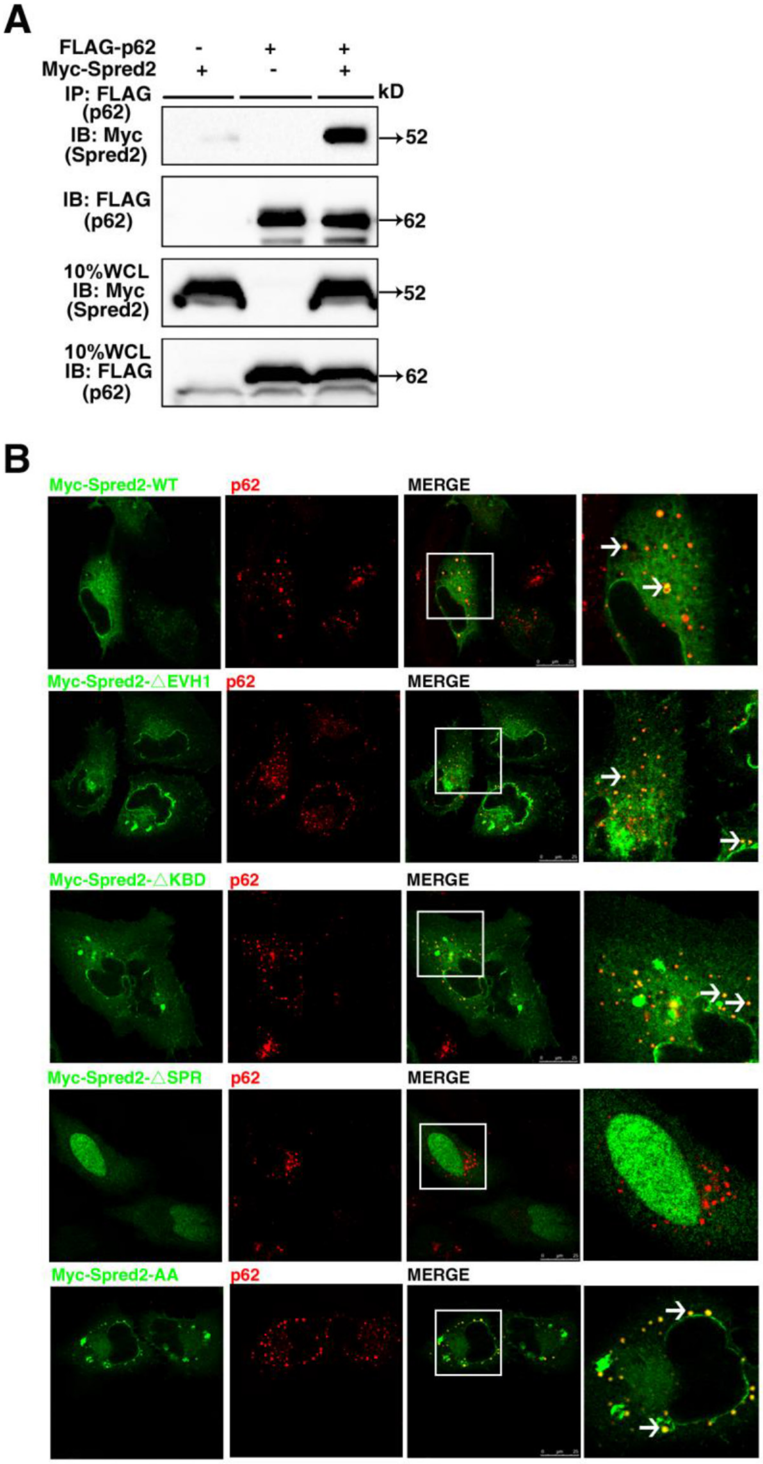

Supplementary Figure S3: Spred2 interacts and co-localizes with p62. (**A**) 293T cells were co-transfected with Myc-Spred2-WT or vector, in addition to FLAG-p62 or vector, whole-cell lysates were used for IP with an anti-FLAG antibody. (**B**) HeLa cells were transfected with Myc-Spred2-WT, various Spred2 deletions constructs or mutants. The co-localization of Myc-Spred2 and endogenous p62 was analyzed by confocal microscopy. The merged region enclosed within the white square has been enlarged in the right panel for clearer appreciation and the arrows indicate the regions of merge. Scale bars represent 25 µm. All experiments in this figure were performed three independent times.

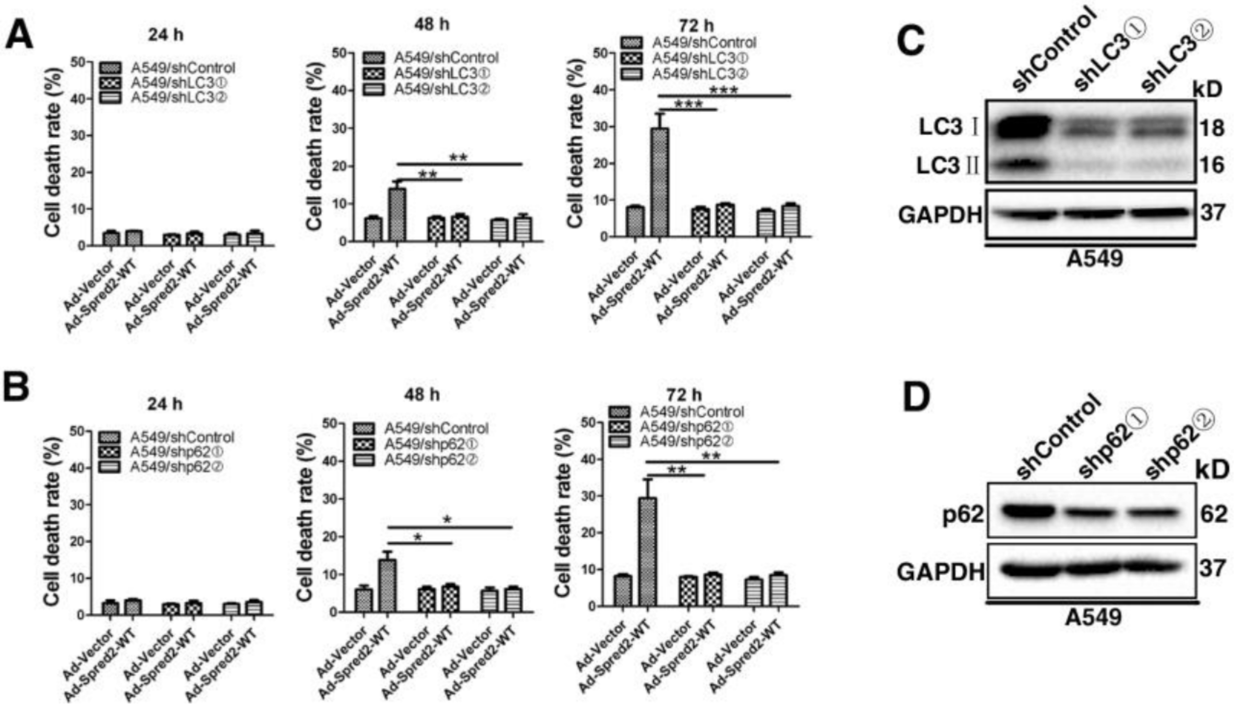

Supplementary Figure S4: Depletion of LC3 or p62 impede Spred2-mediated cell death in A549 cells. Cells with a stable knockdown of LC3 (A549/shLC3), p62 (A549/shp62) or control cells (A549/shControl) were infected with adenoviruses expressing Myc-Spred2-WT or vector control at a multiplicity of infection of 250 for 8 h (**A**, **B**). A549/shControl or A549/shLC3 (A) and A549/shControl or A549/shp62 (B) were determined by cell counting using trypan blue exclusion staining for cell death analysis. (**C**, **D**) Cell lysates of A549/shLC3 cells or A549/shControl cells (**E**) and A549/shp62 cells or A549/shControl cells (F) were analyzed by IB to determine the efficiency of the LC3 or p62 knockdown. Data are represented as mean ± S.D from three independent experiments (*$p < 0.05$, **$p < 0.01$, ***$p < 0.001$).

Supplementary Table S1: Antibodies used in this study

Antibody	Produced in	Dilution	Corporation, CAT number
Anti-Spred2	rabbit	IB: 1:1000	Sigma, S7320
Anti-Spred2	rabbit	IF: 1:100<br>IP: 8 µg/mL	Santa Cruz, sc-98291
Anti-LC3	rabbit	IB: 1:1000	Sigma, L7543
Anti-LC3	mouse	IP: 10 µg/mL	MBL, M152-3
Anti-p62 (SQSTM-1)	rabbit	IB: 1:4000	Abcam, ab109012
Anti-p62 (SQSTM-1)	rabbit	IF: 1:5000	Sigma, P0067
Anti-ATG5	mouse	IB:1:500	Santa Cruz, sc-133158
Caspase-3	rabbit	IB: 1:1000	Cell Signaling Tech, 9662
PARP	rabbit	IB: 1:1000	Cell Signaling Tech, 9532
LAMP2	rabbit	IF: 1:1000	Abcam, ab25631
Anti-ATG16L1	mouse	IF: 1:100	Santa Cruz, sc-393274

GAPDH	rabbit	IB: 1:10000	Proteintech, 10494-1-AP
β-Actin	mouse	IB: 1:10000	Sigma, A1978
Anti-Myc	mouse	IB: 1 μg/mL<br>IP: 8 μg/mL	Invitrogen, 132500
Anti-Myc	mouse	IB: 1:1000<br>IF: 1:5000	Cell Signaling Tech, 2276
Anti-Myc	rabbit	IB: 1:1000<br>IF: 1:250	Cell Signaling Tech, 2278
Anti-FLAG	mouse	IB: 1:4000<br>IP: 4 μg/mL<br>IF: 1:250	Sigma, F3165
Anti-FLAG	rabbit	IB: 1:4000<br>IF: 1:250	Sigma, F7425
Anti-GFP	mouse	IB: 1:4000	Proteintech, 50430-2-AP
Anti-mouse, Alexa 488	Goat	IF: 1:1000	Invitrogen, A-11017
Anti-rabbit, Alexa 488	Goat	IF: 1:1000	Invitrogen, A-11070
Anti-mouse, Alexa 568	Goat	IF: 1:1000	Invitrogen, A-11031
Anti-rabbit, Alexa 568	Goat	IF: 1:1000	Invitrogen, A-21069
Anti-rabbit, Alexa 647	Goat	IF: 1:1000	Invitrogen, A-21244

Supplementary Table S2: The effect of the autophagy modulators and Spred2

Effector	Effect	Interpretation
Rapamycin (Rapa)	LC3II up, p62 down	Induction of autophagosome maturation
Bafilomycin (BafA1)	LC3II up, p62 up	Inhibits VATPase to block the fusion of autophagosome and lysosome
Chloroquine (CQ)	LC3II up, p62 up	Elevates lysosomal pH to inhibit the fusion of autophagosome and lysosome
Overexpression-Spred2	LC3II up, p62 down	Enhances the maturation of autophagosome
Knockdown-Spred2	LC3II up, p62 up	Blocks the maturation of autophagosome
